# Psychological Spectrum Experienced by Heart Failure Patients After Left Ventricular Assist Device Implantation

**DOI:** 10.7759/cureus.9671

**Published:** 2020-08-11

**Authors:** Nkechi A Okam, Wiqas Ahmad, Dibyata Rana, Chenet Torrilus, Nusrat Jahan, Surik Sedrakyan

**Affiliations:** 1 Internal Medicine, California Institute of Behavioral Neurosciences & Psychology, Fairfield, USA

**Keywords:** depression in lvad patient, psychological stress in lvad patients, heart assist device and depression, heart assist device (psychology)

## Abstract

Depression and anxiety disorders are prevalent in patients with heart failure. They are associated with adverse effects such as rapid disease progression, poor medication compliance, low quality of life and increased mortality rate. The current literature review aims to provide an overview of the overall rate of depression in patients who receive left ventricular assist device (LVAD) implantation and identify the psychological phases that these individuals experienced peri- and post-LVAD implantation. A PubMed search using regular and Medical Subject Headings (MeSH) keywords identified 239 articles. After applying inclusion/exclusion criteria, removal of duplicate studies, and careful review of articles, 40 studies provided relevant information on our primary end-point. These 40 studies selected include 13 paid articles with abstracts and 27 free full-text articles comprising eight prospective cohort studies, five retrospective cohort studies, six cross-sectional studies, one qualitative study, one randomized clinical trial, one systematic review, four literature reviews, and one practice guide. Our review shows that patients experienced different psychological phases after LVAD implantation. However, as the time from implantation progressed, these patients showed a significant improvement in depression, anxiety, and health-related quality of life.

## Introduction and background

Over the years, heart failure has garnered multiple definitions, most of which explicitly define it as a clinical syndrome characterized by a variety of symptoms (shortness of breath, lower limb swelling, weakness) and signs (elevated jugular venous pressure, pulmonary congestion, peripheral swelling) caused by structural and functional cardiac abnormality resulting in reduced cardiac output and elevated intra-cardiac pressures [1]. Heart failure cases are estimated to rise to eight million by 2020 in the United States [2]. The disabling nature of heart failure is commonly associated with depression. The prevalence rate of depression in patients with heart failure is about 20%-40%, which is 4%-5% higher for major depression in the general population [3]. Depression in patients with heart failure results in low quality of life, poor self-care, increased use of medical resources, and high readmission rate and mortality rate [4]. To enhance the outcome in advanced heart failure patients, who are unresponsive to medical management, left ventricular assist device (LVAD) has become a therapeutic choice. LVADs may serve as a bridge to heart transplant or destination therapy for poor transplant candidates. The goal for implanting an LVAD is to improve the overall quality of life of patients with advanced heart failure; studies have consistently shown that patients' quality of life is significantly enhanced post-LVAD implantation [5,6].

The symptoms of advanced heart failure can mask the symptoms of depression as they both have certain similarities (i.e., sleep disturbances, fatigue, or low energy). This can represent a diagnostic challenge in differentiating the typical symptoms of heart failure against those of depressed patients [7]. Multiple symptoms caused by heart failure can create a burden on the psychological health of patients. The mental stress these patients experience can easily remain undiagnosed due to their reluctance to disclose emotional stress out of fear of being labeled mentally ill; also, health professionals focus more on the treatment of heart failure symptoms and may write off depressive symptoms as a normal response to heart failure [8-12]. Ultimately, depression manifests itself through physical symptoms such as loss of appetite, weight loss, and sleep disturbance. Thus, most patients consult with other specialists rather than psychiatrists [8-12]. It is of utmost importance to identify heart failure patients with concomitant depression. Only after identifying heart failure patients with comorbid depression will we truly understand the role of LVAD in alleviating or worsening depression in patients with heart failure peri- and post-LVAD implantation. Some recipients of LVAD also describe emotional and psychological stress due to difficulties encountered in the care of the device (e.g., adapting to dependence on a power source and handling of the device), reduced sleep, pain, reduced activities of daily living, and reduced adherence to complex medication regimens [13]. The symptoms experienced by patients after LVAD implantation need to be thoroughly studied to understand if the resultant symptoms are an initial response to major surgery or heart failure depression.

This literature review will compare data from various studies providing an insight into understanding the overall rate of depression in patients who received LVADs, identify the psychological phases experienced by patients post-LVAD implantation, and whether LVAD improves existent heart failure depression or brings about a new-onset depression.

## Review

Using parallel strategies to identify original research and review articles from PubMed, data was collected using Medical Subject Headings (MeSH) and regular keywords. After carefully reviewing titles, abstracts, and free full-text articles, relevant publications and their reference lists were reviewed to identify additional publications. Table 1 presents MeSH and regular keywords used for the literature search.

**Table 1:** MeSH and regular keywords used to find relevant publications appropriate for this literature review LVAD, left ventricular assist device; MeSH, Medical Subject Headings

Keywords and number of studies	
MeSH keyword	Heart assist device (subheading - psychology)
Total records	121
Records selected	60
MeSH keyword	Heart assist device and depression
Total records	31
Records selected	21
Regular keyword	Depression in LVAD depression
Total records	79
Records selected	37
Regular keyword	Psychological stress in LVAD patients
Total records	16
Records selected	10

Studies were selected based on the following inclusion criteria: (1) English publications, (2) studies published within the past 10 years, (3) human subjects above 19 years old, (4) observational studies and clinical trials, including randomized controlled trials, cohort studies, case-control studies, or review articles. However, studies were exempted using the following exclusion criteria: (1) non-English language publications, (2) human subjects below 19 years old, (3) animal studies, (4) case series and meta-analyses. Table 2 presents the total number of studies selected after applying the inclusion/exclusion criteria.

**Table 2:** Total number of studies selected for the review after applying the inclusion/exclusion criteria LVAD, left ventricular assist device; MeSH, Medical Subject Headings

Number of studies after applying the inclusion/exclusion criteria	
MeSH keyword	Heart assist device (subheading - psychology)
Total records	121
Inclusion/exclusion criteria	
Published within 10 years	86
Patient age ≥19 years old	61
Humans	60
English publication	60
MeSH keyword	Heart assist device and depression
Total records	31
Inclusion/exclusion criteria	
Published within 10 years	26
Patient age ≥19 years old	21
Humans	21
English publication	21
Regular keyword	Depression in LVAD patients
Total records	71
Inclusion/exclusion criteria	
Publication within 10 years	57
Patient age ≥19 years old	37
Humans	37
English publication	37
Regular keyword	Psychological stress in LVAD patients
Total records	16
Inclusion/exclusion	
Publication within 10 years	14
Patient age ≥19 years old	10
Humans	10
English publication	10

Applying the regular and MeSH keywords using the inclusion and exclusion criteria, 118 articles were selected. After a thorough review of titles, abstracts, and full-text publications, 78 studies, due to the lack of information about the disease of interest (psychological stress in patients with LVAD implantation), article duplicates, case reports, and meta-analysis, were excluded. Finally, 40 publications in PubMed were selected, of which 27 publications were available in full text online and included the following:

- Twenty observational studies of which eight were prospective cohort studies, five retrospective cohort studies, six cross-sectional studies, and one qualitative study [12-31]

- One randomized clinical trial [32]

- One systematic review (n=1887) [33]

- Four literature reviews [7,34-36]

- One practice guideline [1].

**Figure 1:**
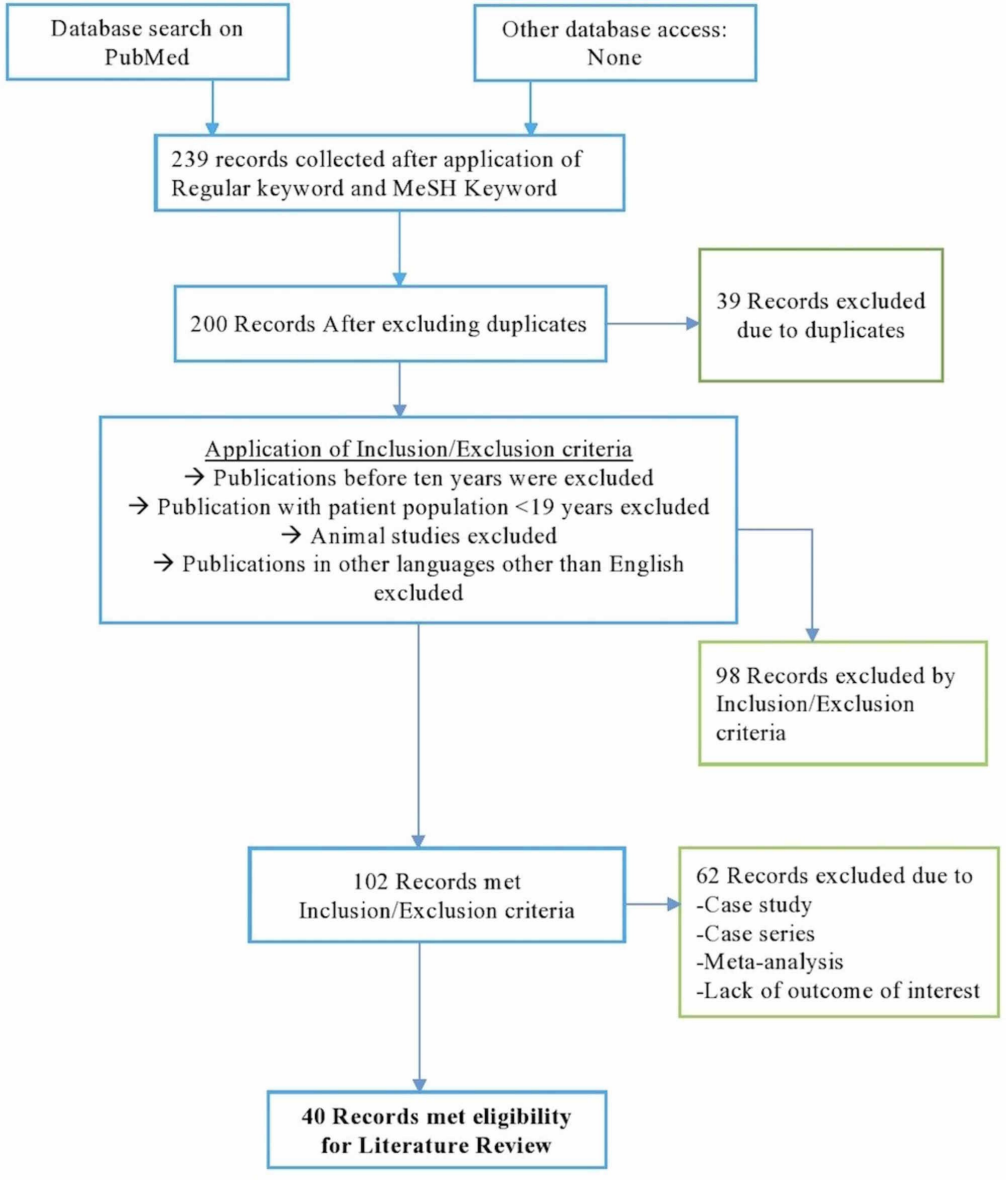
Flowchart representation of the selection process of the current literature review MeSH, Medical Subject Headings

Thirteen paid publications included mostly observational studies and literature reviews [37-49]. After reviewing 27 free available full-text articles, the minimum number of subjects in a study was nine, and the maximum was 1887 [33,49]. In total, the number of subjects in all publications excluding the review article was 4721 [12-33,38,39,41-45,48,49]. Among all 40 studies, 23 studies focused on LVADs' psychological implications in heart failure patients. Two publications also included the psychological experience of secondary individuals, such as spouses and caregivers of LVAD implantation patients explicitly [29,31]. All 27 publications are available for review in the free full-text form on PubMed and citations on information borrowed from the literature are provided. Figure 1 represents a flowchart of the selection process of the current literature review.

Discussion

The analysis conducted was intended to demonstrate the overall rate of depression in patients who received LVAD and identify the post-LVAD psychological phases experienced by these individuals. Advanced heart failure patients may eventually evolve from medical management to requiring a heart transplant. Due to the limited availability of donor heart or weak candidacy, some patients may require a heart assist device either as a bridge to heart transplant or as destination therapy. Most patients with advanced heart failure already experience anxiety, depression, and other mood disturbances, which if diagnosed early can improve outcomes after LVAD implantation. The evaluation of the psychological response LVAD patients experience after implantation has led to the synopsis of four phases. In the pre-LVAD period, patients provide an insight into the limitation brought on by heart failure symptoms and how the desperate need to relieve these symptoms may have influenced their decision on LVAD implantation [35]. The next phase after LVAD implantation is the hospitalization phase [35]. During this phase, patients are highly dependent on the medical team and learn essential skills about the heart device, which can be overwhelming for them [35]. Studies show a high prevalence of adjustment disorder after LVAD implantation [14,22]. During this phase, the anxiety level is amplified, which can be explained by the presence, weight, and sound of the heart assist device. In this phase, patients can also experience disruption in their endocrine, metabolic, cerebral circulation, or adverse effect of medication [14]. The third phase is an early adaptation phase [35]. In the early adaptation phase, patients learn to apply skills learned from the hospital to their everyday routine and modify their home environment to accommodate the electrical necessity of the heart assist device [35]. In this phase, patients are usually anxious as they recognize the need to be attentive as the heart assist device is vital in sustaining their lives [14]. Due to the vulnerability of patients in this phase, caregivers play a huge role in their physical and psychological well-being. Lastly, in the final stage described as a late adaptation phase, the patient becomes accepting of the new normal and show appreciation of the device [35]. In this stage, patients express a sense of increased self-esteem as they understand the complexities of the heart device and how to manipulate it [35]. The duration of these phases can vary as every patient responds differently to stressors.

After an overall view of the selected studies, majority of them showed statistically significant improvement in anxiety, depression, and quality of life after LVAD implantation with some studies identifying changes as early as within 30 days to 12 months after implantation regardless of treatment strategy (bridge to therapy vs. destination therapy). The ability of humans to adapt to changes can explain the findings in the studies. In the past 10 years, multiple research on patients with LVAD implantation has provided more insight into understanding patients' psychological responses to the implantation of a heart device designed to keep them alive. Tables 3, 4 summarize retrospective cohort studies, prospective cohort studies, and systematic reviews on the evolution of ideas on patients' psychological well-being post-LVAD implantation.

**Table 3:** A summary of retrospective cohort studies on the evolution of ideas on patients' psychological well-being post-LVAD implantation LVAD, left ventricular assist device; HTX, heart transplant; HR, hazard ratio; INTERMACS, Interagency Registry for Mechanically Assisted Circulatory Support

Author/date	Study design	Sample size	Main ideas	P-value
Heilmann et al., 2012 [22]	Retrospective cohort study	44	A study on LVAD patients shows 43% of patients with adjustment disorders, 14% with depression, and 18% with multiple diagnoses. Listing for HTX for more than 30 days before LVAD implant does not affect the use of psychotherapeutic support by LVAD patients or the diagnosis of mental illness.	N/A
Snipelisky et al., 2015 [23]	Retrospective cohort study	136	Depression (HR 1.72, 95% CI 1.38-2.15) and illegal drug use (HR 1.67, 95% CI 1.17-2.39) were associated with an increase in the readmission rate. Most common cause of readmission was gastrointestinal bleeding (15.8%), ventricular arrhythmias (12.1%), heart failure (12.0%), driveline-related infection and fracture (8.0%) and hemolysis or LVAD thrombosis (5.2%).	N/A
Kato et al., 2015 [24]	Retrospective study	33	LVAD patients showed a better quality of life at three months and six months than patients with stage D heart failure and extracorporeal LVAD (P<0.05). Patients hospitalized for LVAD-related complications, female sex, and patients with higher levels of anxiety before the operation had lower quality of life.	<0.05
Lundgren et al., 2018 [25]	Retrospective cohort study	258	It was observed that LVAD patients who actively smoked at the time of admission had higher chances of death one year after implantation (P=0.011). LVAD patients with a history of illicit drug use were at risk of readmission (P=0.043). A history of depression (P=0.048) or anxiety (P=0.02) before LVAD implantation was associated with a higher readmission rate.	N/A
Voltolini et al., 2019 [26]	Retrospective cohort study	20	This study, regardless of patient age or preoperative INTERMACS, showed significant improvement in quality of life after two years of LVAD implantation. Indication for LVAD treatment (destination therapy and bridge to transplant) may have impacted the long-term quality of life with destination therapy LVAD patients showing improvement in quality of life from baseline to one year and remained stable two years after implantation. Bridge to transplant LVAD patients also showed significant improvement in the quality of life one year after LVAD implantation, but a subsequent decline back to baseline two years after implantation. Depression and anxiety after two years of study showed statistically significant improvement (P=0.002).	0.002

 

**Table 4:** Prospective cohort studies and systematic reviews on the evolution of ideas on patients' psychological well-being after LVAD implantation LVAD, left ventricular assist device; DT, destination therapy; BTT, bridge to transplant; HTX_prim_, primary heart transplantation; VAD_dest_, ventricular assist device on destination therapy; VAD_htx_, ventricular assist device waiting for transplant; HTX_vad_, successfully bridged to transplant on ventricular assist device; PHQ-9, Patient Health Questionnaire - 9; GAD-7, Generalized Anxiety Disorder 7-Item; KCCQ-CS, Kansas City Cardiomyopathy Questionnaire - Clinical Summary; KCCQ-OS, Kansas City Cardiomyopathy Questionnaire - Overall Summary; NYHA, New York Heart Association; OMM, on medical management

Author/date	Study design	Sample size	Main ideas	P-value
Brouwers et al., 2011 [33]	Systematic review	1887	This systematic review indicates that patients who received LVAD showed improvements in health status, anxiety, and depression in the first 3 months irrespective of the type of LVAD implanted (pulsatile or continuous-flow devices) and clinical setting (DT or BBT). Patients on LVAD reported significant improvement in health status and depressive symptoms compared to medical management alone (P<0.05), but not when compared to a transplant recipient.	<0.05
Heilmann et al., 2011 [14]	Prospective cohort study	51	The most prevalent mood disturbance was adjustment disorders. Two-third of all patients (VAD_dest_, VAD_htx_, and HTX_vad_, or heart transplant for HTX_prim_)suffered significantly from response to severe stress and adjustment disorders (F43.x) and 15% from mild or moderate depression (F32.0 and F32.1). One experienced an episode of anxiety attack (P=0.03).	0.03
Reynard et al., 2014 [15]	Prospective cohort study	66	All Patients displayed significant improvement in depression and anxiety after LVAD implantation that remained stable even through 1 year (P<0.0001). In the cohort study, patients at the time of implantation presented clinically significant symptoms of depression and anxiety identified with a score of 12.1 on PHQ-9 and 10.4 on GAD-7. Furthermore, after LVAD implantation, there was a clinically meaningful improvement in their depressive symptoms.	<0.0001
Brouwers et al., 2014 [16]	Prospective cohort study	54	KCCQ-CS and KCCQ-OS used to determine disease-specific health status overtime showed a significant improvement from baseline to 3 months follow-up. However, the study showed a slight decrease between 6 months and 12 months. Depression, but not anxiety, was a powerful predictor of health status, with an increase of 1 point on depression correlating with a decrease of 1-3 points in health status (P<0.001).	<0.001
Estep et al., 2015 [17]	Prospective cohort study	200	Patients with LVAD had more possibilities of being alive at 12 months with significant improvement in their NYHA class symptoms, depression, and quality of life. However, patients on medical management had less adverse effects when compared to LVAD patients. Patients with LVAD implantation had a high PHQ-9 score at baseline (average 11, signifying moderate depression) vs. patients on medical management who had low baseline score (average 7 meaning mild depression). After 12 months, LVAD patients showed improvement from moderate to mild level of depression while OMM patients remained on mild depression (P<0.001).	<0.001

A randomized clinical trial compared the outcome between women and men using a continuous-flow LVAD as a bridge to transplantation. The study showed that the survival rate between men and women with LVAD was equivalent [32]. The percentage of patients with New York Heart Association (NYHA) functional class I or II symptoms improved from 0% at baseline to 83% for women and 85% for men at six months (P<0.001) [32].

Next are summaries of more prospective cohort studies that provided in-depth insight into the research analogy. Patient-Reported Outcomes Measurement Information System® (PROMIS®) Depression sleep disturbance short form (SF) 8a and Anxiety sleep disturbance SF8a showed patients had a mean t-score of 56 (±5) and 53 (±12), respectively, before LVAD implantation. In contrast, the t-score changed to 44 (±6) and 42 (±9), respectively, 48 weeks after implantation. When plotted on a generalized linear mixed (GLM) model, a decrease overtime signifying improvement with P=0.03 and P<0.001 for depression and anxiety, respectively, was observed [18]. Another study by Shah et al. showed improvement in health-related quality of life (HRQOL), depression, and heart failure symptoms seen in LVAD patients as compared to patients on medical management in the Interagency Registry for Mechanically Assisted Circulatory Support (INTERMACS) 4 group [19]. The two-year survival of LVAD patients, when compared with optimal medical management (OMM) patients in INTERMACS 4 group met all-composite end-point with improvements in (1) New York Heart Association (NYHA ) functional class (≥1 class), (2) EuroQoL (EQ)-5D visual analog scale (VAS) (>20 points), and (3) Patient Health Questionnaire - 9 (PHQ-9) score (≥5 points) (P<0.001) [19]. In a study by Lee et al., during the follow-up period of 6 months, LVAD patients in both patient groups (destination therapy and bridge to transplant) showed similar significant improvements in HRQOL, and significant improvement in shortness of breath, while moderate to significant improvements in wake disturbances [20]. A similar study, also conducted by Lee et al., showed significant improvement in shortness of breath (<0.001) and fatigue within 30 days after LVAD implantation, which was followed by subsequent improvement through 180 days [21]. During the study, it was found that heart failure causes the cardiac muscle to stretch, which led to the elevation of serum N-terminal pro-B-type natriuretic peptide (NTproBNP) [21]. The implantation of LVAD led to a reduction in myocardial stretching and improvement in cardiac output. Thus, changes in myocardial stretching, which was measured by NTproBNP, were associated with an enhancement in shortness of breath, fatigue, cough, early satiety, and depression [21]. The findings in each study are crucial in the management of patients with LVAD implantation. It is essential to know the psychological implications exerted by the heart assist device in an individual's life, as a drastic change in one's routine can impact his or her social, physical, sexual, and psychological experience. The most extensive and most current study in our database had a follow-up period of two years after LVAD implantation [26]. Findings in this study are consistent with evidence that LVAD is highly effective in enhancing hemodynamics and improving functional capacity, which resulted in a favorable effect on the aspect of anxiety and depression in the two-year follow-up period. However, due to a lack of improvement in pain, discomfort, and self-care, patients experience difficulties with LVAD management and care, including LVAD tasks and limitations, and device's effect on self-image [26].

Despite a decrease in depression and anxiety in the majority of studies, adjustment disorders were found in 43%, depression in 14%, and multiple diagnoses in 18% LVAD patients [22]. Symptoms of depression and anxiety can be debilitating, leading to an increase in medication non-compliance, hospital readmission, higher medication cost, higher mortality rate, and decline in physical health [33]. Thus, early identification is necessary as the incorporation of psychological support as a significant part of their treatment plan might be lifesaving. It is clinically and statistically significant to assume that improvement in LVAD patients' mental well-being reflects on their quality of life as studies show depression in these patients to be a powerful predictor of their quality of life [16,28,30]. Partners of patients who received LVAD implantation reported higher levels of psychological breakdown, anxiety, and depression than LVAD patients [29]. They, therefore, stand a chance to gain from psychological therapy. An exciting find was in a study that measured saliva cortisol as a physiological response to stress in patients post-LVAD implantation [28]. This study shows the correlation between psychological and physiological factors in these patients as normal morning saliva cortisol was associated with low levels of depression in the study sample [28].

Limitations

There are limitations to writing a literature review on the psychological effect of LVAD implantation in heart failure patients. The variabilities of heart failure as a subject and relatively limited information on an LVAD can explain these limitations. The target population of choice posed a challenge. Only limited studies were available on patients who received LVAD, and already available reviews had additional variables in their analyses, which affected the literature’s homogeneity. Current research studies restricted their review in terms of gender (no gender-specific research) and length of follow-up (no study discussed long-term follow-up beyond two years). Studies used various modalities and criteria to diagnose depression, anxiety, and other mood disorders in LVAD patients. As such, the analysis and comparison of the results of multiple studies presented a challenge.

## Conclusions

The current literature concludes that in the peri-implantation and immediate post-LVAD implantation periods, patients experienced depression, anxiety, and mood disorders. However, as time elapsed, these patients passed through psychological phases of various lengths where they learned to adapt to changes, and shortly after, the psychological burden brought on by the heart assist device improved exponentially. This review should prompt researchers to conduct lengthier studies that are gender specific and explore more on the psychological phases experienced after LVAD implantation. Researches should provide knowledge on how medical management can influence each psychological phase and if medical intervention can shorten the duration of these phases and optimize LVAD patients' quality of life.
